# Supplementary material for: Structural heterogeneity of α-synuclein fibrils amplified from patient brain extracts
Source: Nat Commun. 2019 Dec 4;10:5535. doi: 10.1038/s41467-019-13564-w (PMC6893031; doi:10.1038/s41467-019-13564-w)
Supplement: Supplementary file 1 — Supplementary Information [file 41467_2019_13564_MOESM1_ESM.pdf]

Supplementary Information

Structural heterogeneity of α-synuclein fibrils amplified from patient brain extracts

Timo Strohäker et al.

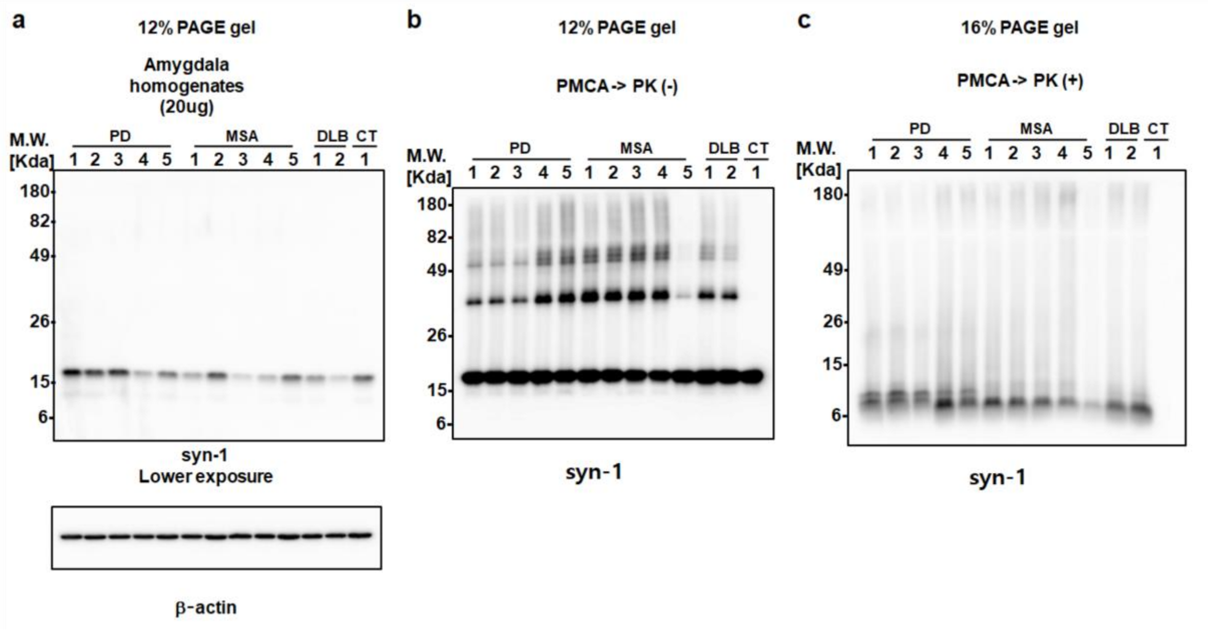

Supplementary Fig. 1 Amplification of aSyn fibrils from brain extracts. a, Immunoblotting of brain extracts (CT; brain extract from an individual, in which a synucleinopathy was excluded). The degree of aSyn expression level was not directly correlated with synucleinopathies. **b,** Immunoblotting of PMCA products with indicated brain extracts as seeds. High-molecular weight aSyn was induced through PMCA with patient brain extracts, whereas control brain extract failed to induce high-molecular weight aSyn after PMCA. **c,** Immunoblotting of PMCA products with proteinase-K (PK) digestion. PK-resistant signals were detected in samples from patients pathologically confirmed with a synucleinopathy, whereas nothing remained in the control sample.

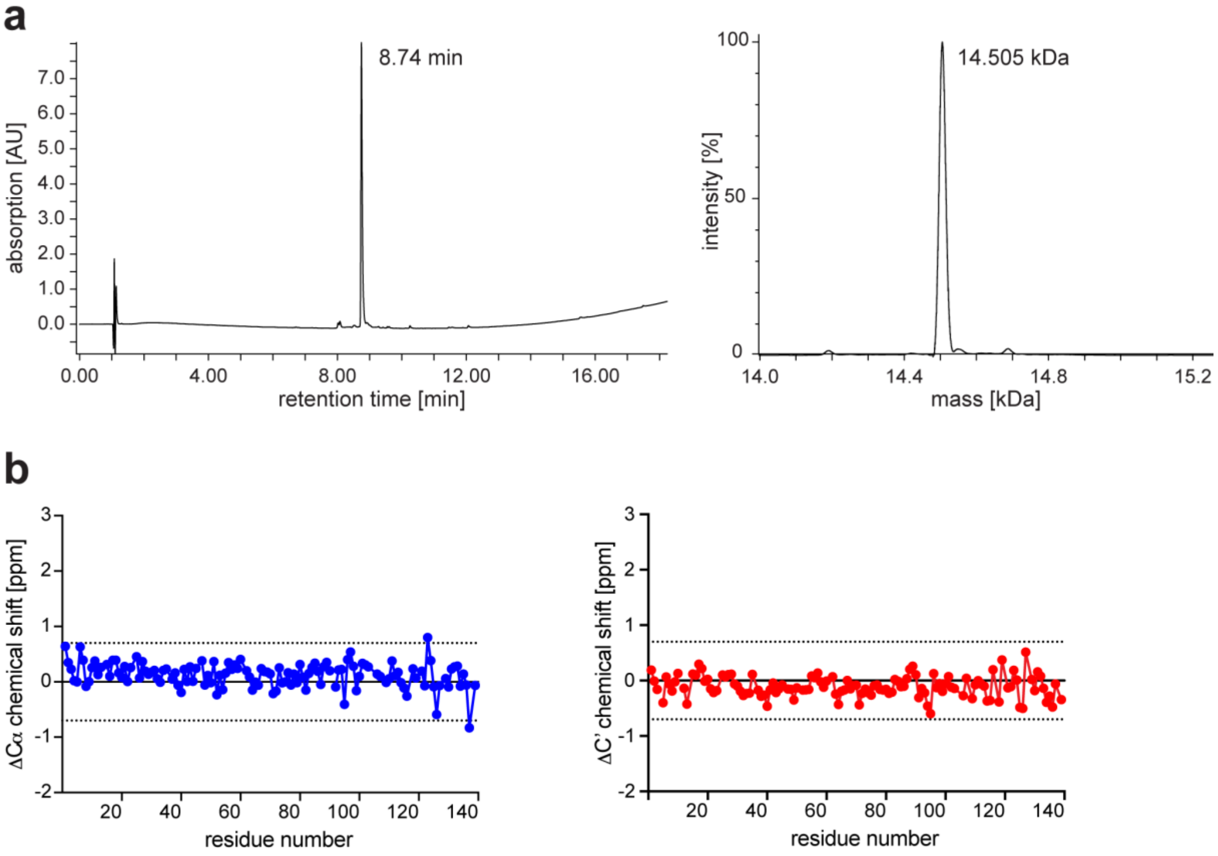

Supplementary Fig. 2 aSyn is disordered in fibril dissociation buffer. a, Mass spectrum of N-terminally acetylated aSyn. **b,** Cα and C' secondary chemical shifts of N-terminally acetylated aSyn in the dissociation buffer (4M GdSCN, 0.4 % formic acid, pH 2.5). Because secondary chemical shifts are small, aSyn does not contain hydrogen-bonded secondary structure in the fibril dissociation buffer.

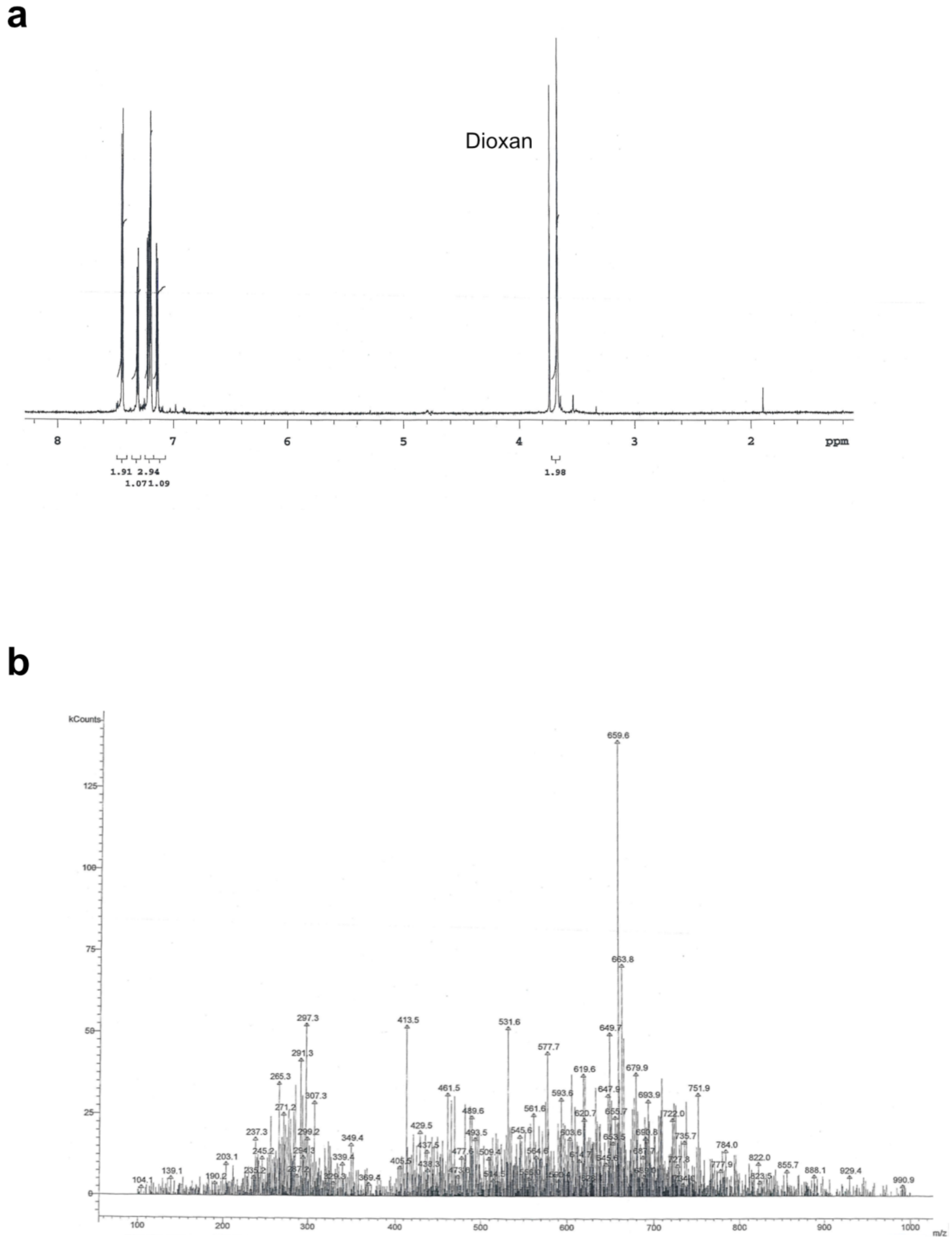

Supplementary Fig. 3 ^{1}H NMR spectrum (a) and mass spectrum (b) of HS-68.

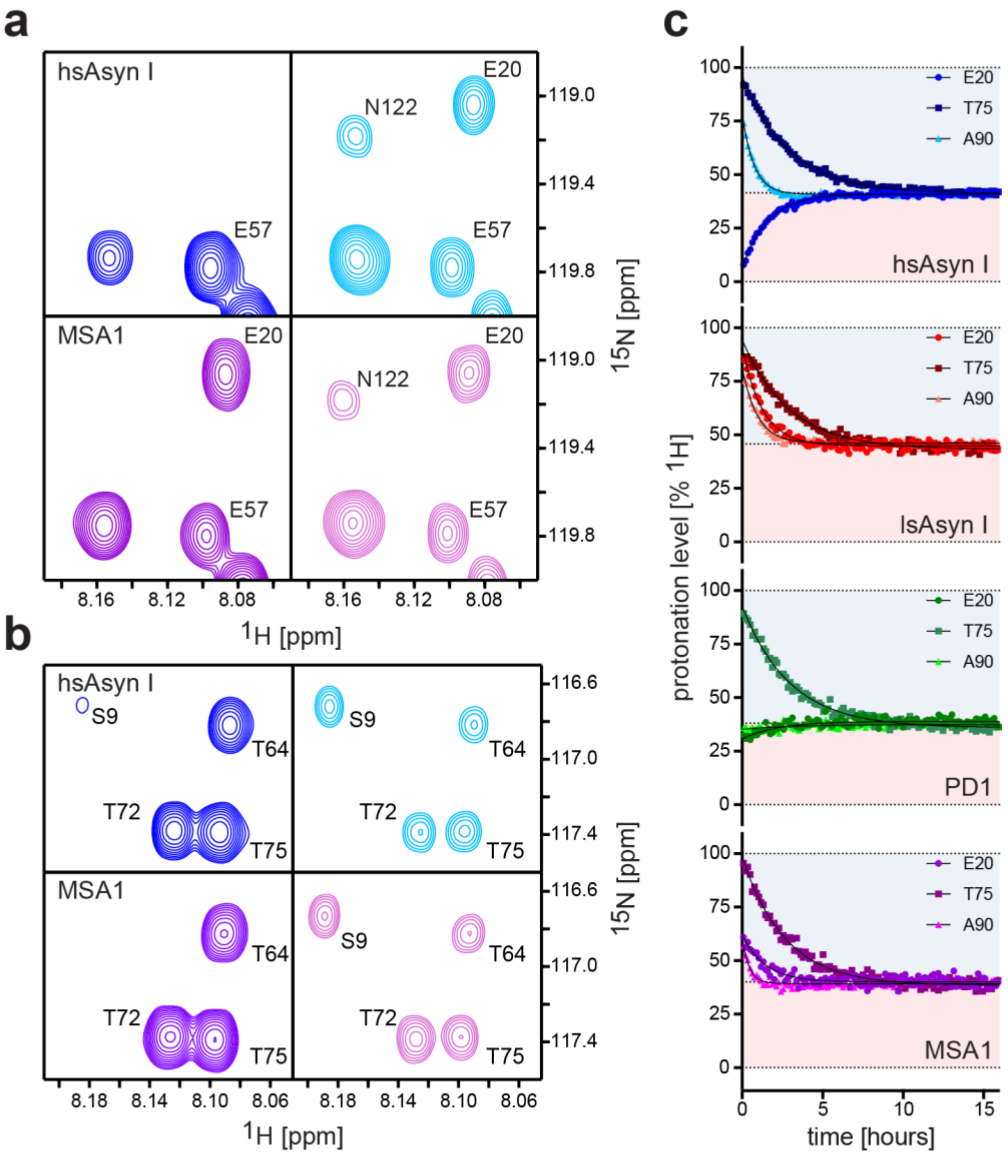

Supplementary Fig. 4 HD exchange NMR data of different aSyn fibrils. a,b, Selected regions from ^{1}H-^{15}N correlation spectra recorded after fibril dissociation at the beginning (left) and end (right) of the back-exchange period for the hsAsyn polymorph (top) and aSyn fibrils amplified from the brain extract of a MSA patient (bottom). **c,** Time-dependent back exchange of selected residues for different aSyn fibril types (from top to bottom; Table 1): hsAsyn, lsAsyn, PD1, MSA1.

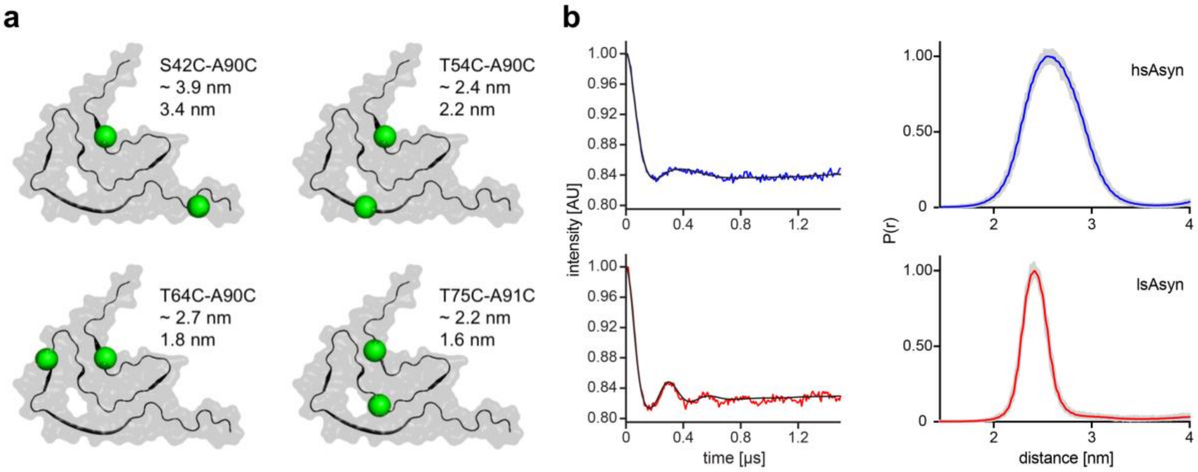

Supplementary Fig. 5 EPR PELDOR measurements. a, Distances measured by PELDOR for four MTSL-pairs in the *in vitro* high-salt polymorph of aSyn (upper values; Pornsuwan et al. *Angew Chem* 2013) in comparison to the cryo-electron microscopy structure (lower values; Cα-Cα distances in PDB code: 6A6B). Changes in the orientation of the MTSL-side chain were previously estimated to amount to ~0.4-0.5 nm, so could reach ~1 nm for a MTSL-pair. **b,** Dipolar modulation (left; corrected for background; fit in blue/red) and normalized distance distributions (right) determined by artificial neuronal network analysis implemented in DeerAnalysis2018 for hsAsyn (top, blue) and lsAsyn (bottom, red).

aSyn T54C mutation forward primer
5'-GAG TGG TGC ATG GTG TGG CAT GCG TGG CTG AGA AGA CCA AAG-3'
aSyn T54C mutation reverse primer
5'-CTT TGG TCT TCT CAG CCA CGC ATG CCA CAC CAT GCA CCA CTC-3'
aSyn A90C mutation forward primer
5'-GGA GCA GGG AGC ATT GCA TGT GCC ACT GGC TTT GTC AAA AAG-3'
aSyn A90C mutation reverse primer
5'-CTT TTT GAC AAA GCC AGT GGC ACA TGC AAT GCT CCC TGC TCC-3'

Supplementary Table 1 QuikChange Primer.
